# Metallization of 3D Printed Polymers and Their Application as a Fully Functional Water‐Splitting System

**DOI:** 10.1002/advs.201801670

**Published:** 2019-01-24

**Authors:** Xinran Su, Xinwei Li, Chun Yee Aaron Ong, Tun Seng Herng, Yanqing Wang, Erwin Peng, Jun Ding

**Affiliations:** ^1^ Department of Materials Science and Engineering National University of Singapore Singapore 117576 Singapore

**Keywords:** 3D printing, electrochemical full cells, electroless plating, polymers, water splitting

## Abstract

In this work, the plating of high‐quality amorphous nickel–phosphorous coating with low resistivity of 0.45 µΩ m (298 K) on complex 3D printed polymeric structures with high uniformity is reported. Such a polymer metallization results in an effective conductivity of 4.7 × 10^4^ S m^−1^. This process also allows flexible structures to maintain their flexibility along with the conductivity. Octet‐truss structures with nickel–iron‐(oxo) hydroxide nanosheets electrodeposited onto further displays excellent water‐splitting performance as catalytic electrodes, i.e., in KOH (1 m, aq), a low oxygen evolution reaction (OER) overpotential of 197 mV at 10 mA cm^−2^ and Tafel slope of 51 mV dec^−1^. Using this light‐weight electrode with high specific area, strength, and corrosion resistance properties, a fully functional water‐splitting system is designed and fabricated through the concentric integration of 3D printed components. A dense polymeric mesh implemented is also demonstrated as an effective separator of hydrogen and oxygen bubbles in this system.

Polymeric materials find diverse applications; from daily necessities to high end uses such as in aerospace, automobile, and healthcare, due to their desirable properties such as light weight, low cost, ease of manufacturing, and adaptive mechanical properties—from being flexible and stretchable to rigid with high hardness and strength. For a modern‐day polymeric product design associated with complexity and integration, conceptually new methods which are scalable, sustainable, and resource efficient will be needed. 3D printing is one such rapidly growing disruptive manufacturing technique capable of extreme design freedom and prototyping flexibilities with the guaranteed part quality for polymeric fabrication. As such, 3D printed polymers find applications in various fields, i.e., actuators, sensors, antennas, biomedical devices, etc.^1^ Despite this, one drawback of 3D printed polymers is their lack of electrical conductivity; hampering their potentials for usage as electrical conducting materials. The importance of “metallization” of polymers is apparent, given the requirements of modern‐day product design; ongoing research studies on making polymers conductive are prevalent. Many works had focused on the incorporation of conductive components to a 3D printable polymer matrix. Nanostructured carbon‐based fillers were widely studied^2^ with a range of final electrical conductivities varying from 1.6 × 10^−2^[[qv: 2a]] to 81 S m^−1^.[[qv: 2c]] Nanostructured metal‐based fillers were also studied with optimized conductivity reported as 9.1 × 10^−4^ S m^−1^.^3^ However, there is an upper limit to achieve higher conductivities due to the associated 3D printing techniques; since increasing filler loadings will lead to problems in filler dispersions and ink/resin^4^ printability. Another approach was the inclusion of ions within hydrogel networks with conductivities reported to be around 2.9 S m^−1^ for the as‐print.[[qv: 2b]] Apart from the limited conductivities which will always be less than their corresponding saturated aqueous salts (already low of ≈10 S m^−1^);^5^ weak mechanical strengths and low working stabilities in air from the hydrogel matrix also impede usage for actual applications.

To overcome such challenges, surface functionalization can be considered instead, i.e., deposition of conductive layers, for example—metallic films, directly on polymers. It has been widely adopted for the fabrication of mechanical metamaterials with radical properties such as being strong and ultralight, recoverable, multiscale, and hierarchically architectured.^6^ However, no work had yet been reported on the electrical performance and potential applications as catalysts for such metallic deposited polymers to our best knowledge. Herein, we present 3D printed polymeric structures metallized by electroless plating with the selection of amorphous NiP. Such a method allows an areal selective, facile, low‐cost, and fast route to achieve conductivities in polymeric structures (with an effective overall conductivity >10^4^ S m^−1^) with yet mechanical strengthening. Uniform plating on complex interior geometries was also made possible with the addition of surfactants for the aqueous processing solutions. In addition, the NiP coating is also extremely robust, given its excellent adhesion with polymer substrates and yet still allowing flexibilities of soft polymers. It also has excellent corrosion resistance from the amorphous nature of NiP,^7^ ideal for actual applications in chemically harsh environments. As a proof of concept, we next will showcase NiP metallized polymers for an application in water splitting.

Seeking an alternative fuel source, much research have been put into the developments of sustainable advanced catalytic materials and electrode architectures to overcome the thermodynamical and kinetical challenges brought about by the production of H_2_ gas from H_2_O.^8^ Among them, functionalized 3D printed electrodes had shown to be promising candidates for their designable porous architectures, excellent mechanical integrity, and facile synthesis routes.^9^ In this work, an efficient 3D printed polymeric octet‐truss electrode is made active by electrodeposition of complexes synthesized from earth‐abundant Ni and Fe elements onto its NiP metallized surface; capable of achieving a low oxygen evolution reaction (OER) overpotential of 197 mV (10 mA cm^−2^, KOH (1 m, aq)) with a Tafel slope of 51 mV dec^−1^. Further, we demonstrate the integration of such an electrode into a similarly 3D printed, compact, fully functional water‐splitting system capable of high catalytic efficiencies, gas separation, and gas collection. With this, we illustrate the metallization of polymeric 3D structures, further surface functionalization using economic catalytic material (here nickel–iron‐(oxo) hydroxide nanosheets), study of their electrochemical performance for water splitting, and a demonstration for a 3D printed fully functional water‐splitting system.

To demonstrate the extreme geometrical allowance of 3D printing and the versatility of electroless plating on a variety of materials and surface types, we showcase different NiP plated structures made using different polymers (Experimental Section). Digital images of as‐printed and plated are shown in **Figure** **1**a,b respectively. For electroless plating of NiP, whereby aqueous solutions are employed, the key on plating quality lies on the interfacial properties between the polymer and catalytic solution. Given the low wettability (contact angle ≈ 90°) of, i.e., our used polymer, gas bubble that hinder the plating process form easily in the porous geometry (Figure 1ci and Figure S1a, Supporting Information). In addition, the reaction processes create nanobubbles adsorbed onto the polymeric surfaces that result in unplated pits too (Figure S1b, Supporting Information).^10^ A surfactant (sodium dodecyl sulfate), which can help to reduce the interfacial energy between porous structures and aqueous solutions, was then introduced to eliminate bubbling effects. Such a method is also cheap and nondestructive as opposed to processes associated with surface etching.^11^ As shown in Figure 1cii, a decreased contact angle of ≈29° is observed along with the elimination of entrapped bubbles. Good conductivity and the light weight of such metallized polymers can be seen from Figure 1d, in which an octet‐truss lattice (of truss diameter to unit cell length ratio 1:5) can be upheld freely with two lit light‐emitting diode (LED) wires. Excellent adhesion of as‐plated NiP layer is demonstrated in Figure 1d; under ≈100 cycles of compression for a plated flexible polymer, it can be noted that the layer is still intact to allow conductivity. Harsh compressions and further illustrations can be seen from Movie S1 in the Supporting Information.

**Figure 1:**
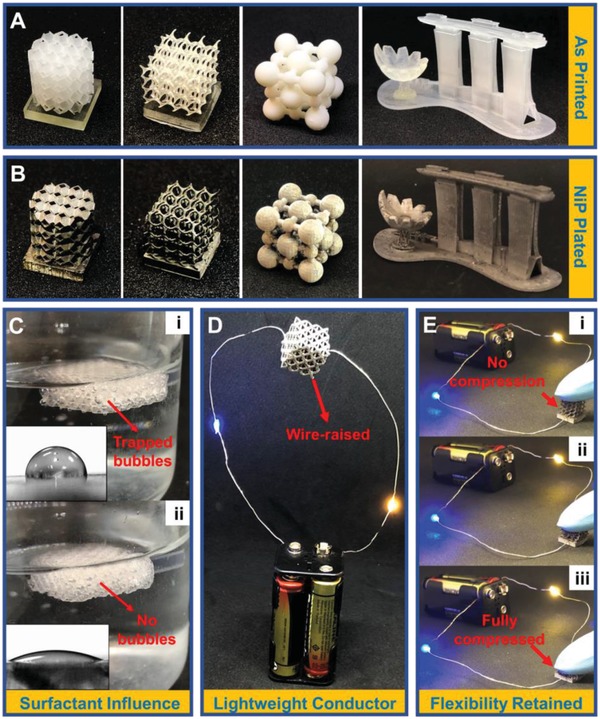
a) 3D printed polymeric objects using different materials and techniques; b) their NiP plated counterparts. c) i) Low wettability and subsequently entrapped bubbles in the truss electrode, ii) improved wettability and elimination of entrapped bubbles after addition of surfactants. d) Compression of a NiP plated flexible polymer lattice; LEDs shown to be lit throughout 100 cycles. e) Lightweight lattice shown to be upheld freely in air and with high conductivity to light up to two LEDs connected in series.

An optimized plating duration of 30 min was chosen for subsequent studies based on the uniformity and adhesion of NiP layer on the polymer (Figure S2, Supporting Information). Scanning electron microscope (SEM) observation of coating morphology reveals the thickness to be ≈2.7 µm (**Figure** **2**a) with smooth and continuous grains in the order of 1–2 µm (Figure 2b). Such an orderly arrangement ensures favorable electrical conductivity; resistivity of amorphous NiP was measured to be as low as 0.45 µΩ m (at 298 K) with a typical behavior of an amorphous alloy since resistivity decreases much slower with decreasing temperature (Figure 2c), compared to that of a crystalline metal.^12^ Our resistivity compares well with other reported Ni‐based platings.^13^ It is also interesting to evaluate the conductivity of the resultant NiP@Polymer structure (normalized with the polymeric volume). For NiP plated onto a polymeric strut of 500 µm diameter (as used in our water splitting), the structure has an effective conductivity of 4.7 × 10^4^ S m^−1^, which is still much higher compared to those structures reported by 3D printing in the current literature.

**Figure 2:**
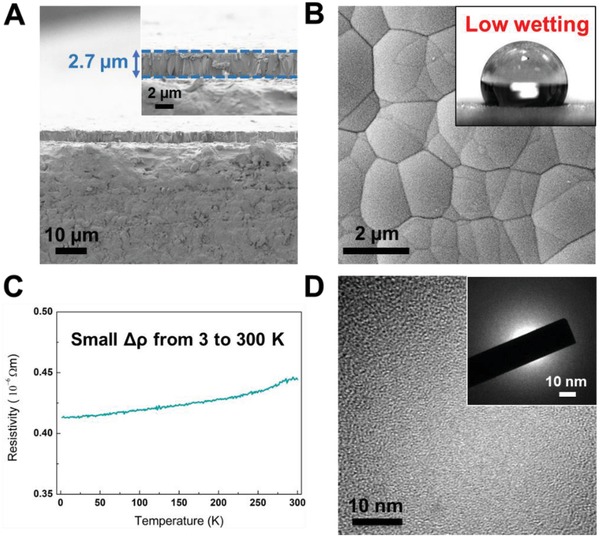
a) SEM images revealing the cross‐section and b) top view of NiP. c) NiP resistivity measured over a wide range of temperatures. d) TEM and SAED revealing the amorphous nature of NiP.

Transmission electron microscopy (TEM) image and the selected area electron diffraction (SAED); inset (Figure 2d), together with X‐ray diffraction (XRD) (Figure S3, Supporting Information); confirm the resultant NiP to be of amorphous nature. Electron dispersive X‐ray spectroscopy (EDX) revealed an averaged atomic ratio of 5:1 for Ni to P. The composition is in the range of amorphous phase,^14^ in agreements with our TEM and XRD findings. X‐ray photoelectron spectroscopy (XPS) (Figure S4, Supporting Information) reveals distinct Ni 2p and P 2p peaks for NiP alloy and Ni^2+^ 2p peaks for oxidized Ni, consistent with results from other work.^15^ The amorphous state of NiP alloys allows its high corrosion resistance,^7^ ideal for applications in chemically harsh environments.

Build upon the success of polymer metallization via NiP plating, we further explored the possibilities for a water‐splitting application using the combination of derived excellent electrical conductivity, high design freedom of 3D printed structures, and further surface functionalization. For structural concern, truss architectures with complex and small internal geometries were considered; in particular, the octet architecture was chosen in this work for its combination of high specific surface area and strength.^16^ A comparison of octet and other popular truss architecture can be found in Table S2 in the Supporting Information; studies reveal superior metrics with 4.1 times increased surface area from a solid bulk. An illustration of its mechanical strength is as shown in Figure S5a and Movie S2 in the Supporting Information. Given a compressive strength of ≈5 MPa, it means that this structure with a density 0.4 g cm^−3^ (2.5 times lighter than water) can withstand up to ≈110 kg of weight before failure initiates for a normal cross‐sectional area of 2.25 cm^2^. This strength to density ratio falls in the upper‐right region of rigid polymer foams (Figure S5b, Supporting Information). Made electrical conductive after uniform NiP coating, (**Figure** **3**e and Figure S5, Supporting Information), such a structure was shown to be suitable as the rigid and lightweight electrochemical substrate for further surface functionalization using the low‐cost, earth abundant, and yet high performing nickel–iron‐(oxo) hydroxides nanosheets (NFNS).^17^ NFNS was electrodeposited (Experimental Section) uniformly on top of the NiP conductive layer and observed to be honeycomb structured (Figure 3b). Figure 3c and Figure S3 in the Supporting Information reveal the NFNS to be of amorphous nature based on SEM, TEM, and XRD examinations. XPS analysis (Figure S7, Supporting Information) confirms their existence and chemical formula approximately to be Ni_2_Fe(OH)_7_ from fit Ni^2+^, Fe^3+^, and O peaks.[[qv: 17b]] In contrast to NiP, NFNS instead displays super hydrophilicity (Figure 3b; inset) which is favorable for water splitting. According to the theory of surface tension, high wettability allows easier gas bubble detachment since electrolyte replacement will be favored.^18^ The NFNS catalytic layer did not affect the resistivity of the coating layer significantly (Figure S8, Supporting Information).

**Figure 3:**
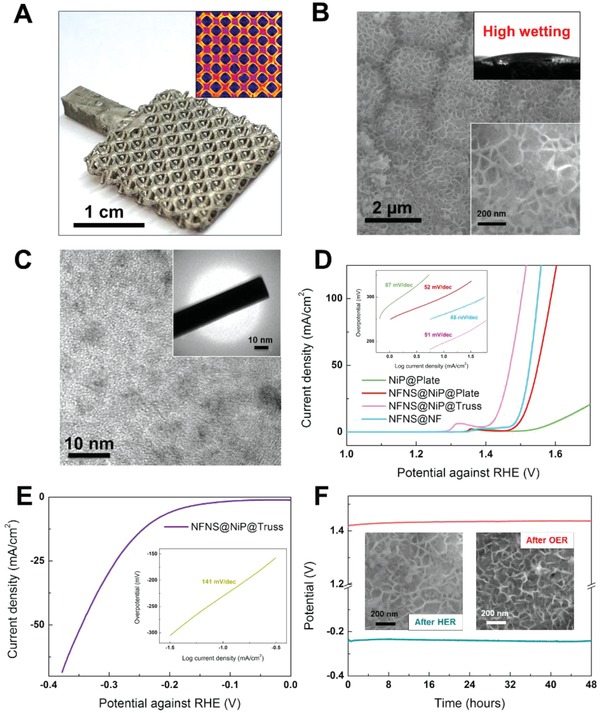
a) Digital image of the NiP plated electrode with the computer‐tomography (inset) revealing uniform coatings throughout the structure. b) SEM image of NFNS; insets reveal the honeycomb like structure and high wettability. c) TEM and SAED reveal the amorphous nature of NFNS. Representative LSV polarization curves on d) OER of NiP@Plate, NFNS@NiP@Plate, NFNS@NF, and NFNS@NiP@Truss, e) HER of NFNS@NiP@Truss sample. f) 48 h stability run on NFNS@NiP@Truss for both OER and HER; insets reveal the SEM morphology after stability run.

Advantages of the structural and material improvements had been evaluated through the performance in OER polarization using four different electrodes—3D printed plate with NiP coating only (NiP@Plate), 3D printed plate with NiP coating and further NFNS deposition (NFNS@NiP@Plate), nickel foam (NF) deposited with NFNS (NFNS@NF), and 3D printed octet‐truss lattice with NiP coating and further NFNS deposition (NFNS@NiP@Truss). As shown in Figure 3d, order of progressive improvements in performance in terms of a lower overpotential goes from NiP@Plate to NFNS@NiP@Plate to NFNS@NF to NFNS@NiP@Truss. With solely NiP plating, NiP@Plate demonstrates a high OER overpotential of 300 mV at 10 mA cm^−2^ with a high Tafel slope of 87 mV dec^−1^. The deposition with NFNS resulted in both the reduction of overpotential and Tafel slope (273 mV and 52 mV dec^−1^ respectively) for NFNS@NiP@Plate, confirming the superiority of NFNS as an effective water‐splitting catalyst. Comparing the plate with the truss electrode, our hallmark NFNS@NiP@Truss indeed revealed an even lower overpotential of 197 mV with a Tafel slope of 51 mV dec^−1^. The significant improvements attribute to the larger surface area of the 3D structure and porous designs associated with effective bubble removal.^19^ Nickel foam, a state‐of‐the‐art substrate widely studied in literature was used for benchmarking.^20^ The measured overpotential of 260 mV at 10 mV cm^−2^ pales in comparison to NFNS@NiP@Truss with a 63 mV higher value. In addition, the performance of our NFNS@NiP@Truss compares favorably with both other reported electrodes associated with Ni and Fe complexes as well as other 3D printed electrodes, as illustrated in Table S2 in the Supporting Information. Such a comparison illustrates the potentials of using hierarchically designed 3D printed electrodes as water‐splitting catalysts to replace that made by noble metals. In order to explore the newly developed NFNS@NiP@Truss electrodes for full water splitting, we have also studied hydrogen evolution reaction (HER) under basic environments. In KOH (1 m, aq), HER overpotential of NFNS@NiP@Truss at 10 mV cm^−2^ was found to be 243 mV with a Tafel slope of 141 mV dec^−1^ (Figure 3e). The result indicated that the NFNS@NiP@Truss can be effectively used as bifunctional electrodes under a basic environment. For practical applications, long term catalytic stability is necessary. As plotted in Figure 3f, prolonged water splitting for 48 h showed constant potentials with miniscule changes for both OER and HER; suggesting excellent working stability. In addition, no observable changes in the NFNS morphology were observed for both conditions based on the SEM inserts. Their honeycomb like structures were retained after a long term reaction.

Utilizing NFNS@NiP@Truss as efficient, bifunctional, and mechanically robust electrodes, we had also designed a water‐splitting full cell (WSFC) after the integration of different polymeric components made by 3D printing (**Figure** **4**a). The designed WSFC consists of four parts: an OER truss electrode with walls as the outer shell; HER truss electrode as the core; a semipermeable gas‐bubble separator with HER cap and gas collection guide for insertion in between the OER and HER electrodes; and an OER cap and its gas collection guide to cover the OER electrodes. Each part will be fabricated individually and then assembled concentrically as shown in Figure 4a. Such a concentric design minimizes the separation between OER and HER electrodes for a reduced electrical resistance while maximizing space usage. Working mechanism is illustrated in Figure 4b— effective water‐splitting reactions taking place at concentrically faced electrodes and subsequent H_2_ and O_2_ gas collections.

**Figure 4:**
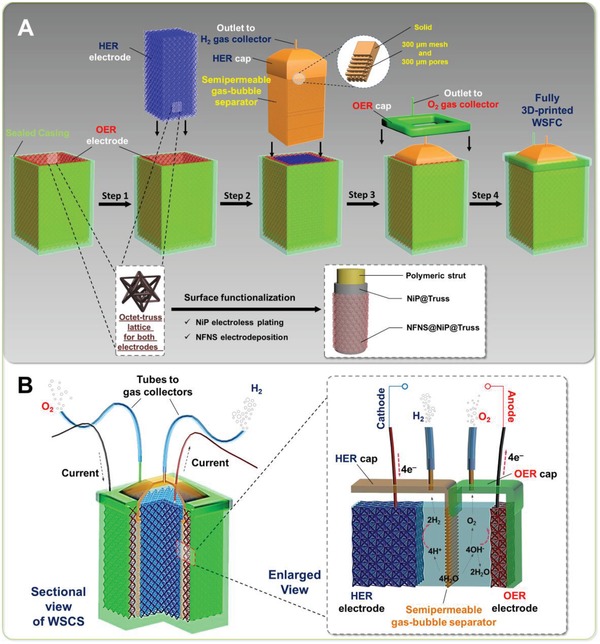
a) Schematics, design, and feature of a water‐splitting full cell (WSFC) through the integration of polymeric components made by 3D printing. b) Sectional view of the as‐assembled and inset shows the working mechanism of water splitting inside the WSFC.

Another key issue for WSFC lies on the separation of H_2_ and O_2_ gases effectively. As such, the semipermeable separating mesh was implemented for this purpose as a gas‐bubble separator. Design (a thin sheet at 50% porosity with 300 µm square pores; Figure S9a,b, Supporting Information) principle is based on maximizing surface areas to physically reflect approaching bubbles while still being porous to allow minimized resistance increase for the electrolyte. As illustrated in **Figure** **5**a, a study of gas bubble motion reveals that visible bubbles formed travelled directly upward toward the electrolyte surface under the influence of buoyancy forces, interacting with the surface and hence creating turbulences that result in bubble mixings. Figure 5b, in turn, shows the effect of our designed separator to block the travelling H_2_ gas bubbles. As seen, bubbles were observed to reflect off the separator surface in a swirling motion with no visible ones observed on the other side of the separator even after a relatively long reaction time of 20 min. This indicates that no (or at least very low quantity) H_2_ gas bubble escaped the dense mesh structure. As shown in Figure S9c, Supporting Information, the separator structure had been further improved by designing the part near the electrolyte surface to be an entirely impermeable solid in order to impede gas bubble exchanges more effectively. Such a designed and 3D printed gas‐bubble separator is a mechanically robust, simple, and effective viable alternative as opposed to polymeric electrolyte membranes.^21^

**Figure 5:**
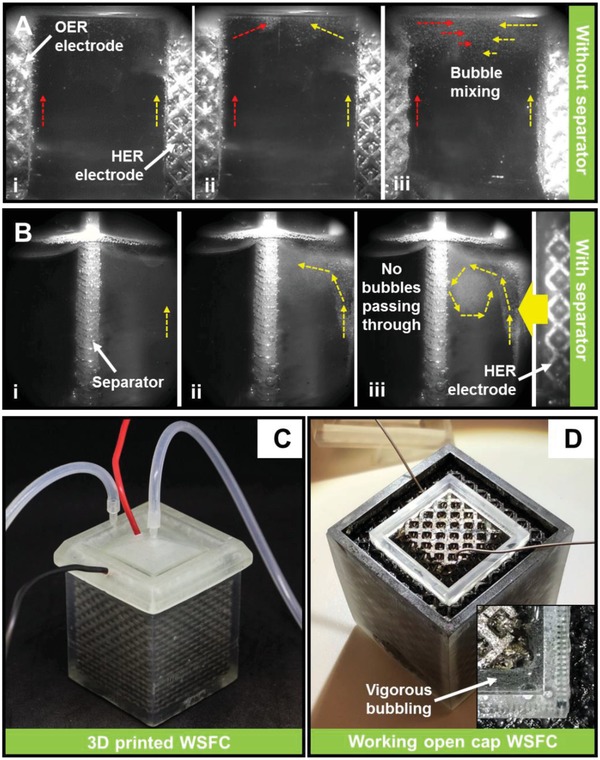
Fast‐speed video snapshots showing bubble motions a) without a separator: i) bubble forming, ii) bubble interacting with surface, and iii) bubble mixing from the turbulence; b) with a separator: i) bubble forming, ii) bubble reflecting off the separator surface, and iii) bubble swirling away from separator. c) 3D printed and assembled WSFC with electrical wires and pipes connected. d) Open‐cap look at the WSFC; inset reveals the vigorous bubbling with three 1.5 V batteries.

Figure 5c shows the digital image of the 3D printed and assembled WSFC. Three batteries with a total voltage of 4.5 V are sufficient to power our WSFC efficiently with vigorous bubbling observed (Figure 5d). The effectiveness of gas‐bubble separator can also be observed with bubbles swirling back to the HER electrode. Mass spectroscopy study further demonstrated a very low content (below detection limit) of H_2_ being found in the collected O_2_ gas. More studies will be required to understand the detailed mechanism in the near future. These fully demonstrate the successful design principles and functionalities of our WSFC.

In conclusion, high‐quality amorphous NiP with low resistivity of 0.45 µΩ m (298 K) was successfully plated on 3D printed polymeric structures of complex geometries. With the success of polymer metallization, an effective conductivity of 4.7 × 10^4^ S m^−1^ can be achieved for our NiP@Truss, higher than that reported previously by other works of 3D printed conductive polymeric structures. It is to note that this plating on a flexible structure allows high conductivity to be retained even after multiple harsh compressions. Further electrodeposition with NFNS on the amorphous NiP layer constituted to a stable NFNS@NiP@truss electrode with promising OER and HER bifunctional catalytic performance, i.e., an OER overpotential of 197 mV (10 mA cm^−2^, KOH (1 m , aq)) and Tafel slope of 51 mV dec^−1^. Our concentrically designed WSFC could work well with three 1.5 V batteries. The gas‐bubble separator implemented was also shown to be highly effective in preventing gas bubble mixings.

## Experimental Section

*3D Printing Materials and Methods*: For samples in Figure 1a, 3D printers and materials used were as follows—Asiga Pico 2 HD (UV 385 nm) digital light projection printer with PlasClear resin for (first from left), FL01 resin for (second), and PlasWhite resin for (third); Form 2 (UV 405 nm) stereolithography printer with RS‐F2‐GPCL‐04 resin used for (fourth). Octet‐truss electrode and WSFC components were fabricated using both Asiga Pico 2 and Form 2 printers. All printers and their material were commercially available.

*Electroless Deposition of NiP Conductive Layer*: Electroless plating of NiP involves two main steps—1) sensitization and activation of the target substrate surface and 2) electroless deposition of NiP. For the sensitization and activation steps, electroless copper solution C (Transene, USA) (HCl 5 wt% and SnCl_2_ 5 wt%) followed by electroless copper solution D (Transene, USA) (HCl 5 wt% and PdCl_2_ 5 wt%) were respectively used to immerse dip the substrate for 5 min each, followed by 60 °C oven dry after each step. The surfactant, sodium dodecyl sulfate (60 × 10^−6^ m) was added into both catalyst solutions at this step. After that, an acid (5% H_2_SO_4_) was used for dipping for 5 min afterward. After the activation step, the substrate was rinsed with deionized (DI) water and thoroughly dried in the oven. Electroless deposition was done in preheated electroless Ni Solution (Transene, USA) (NiCl_2_, NaPO_2_H_2_, C_4_H_4_Na_2_O_4_, HF) of ≈97 °C with the temperature maintained throughout. All used parameters and methods were prior optimized for 3D printed polymer. A suitable deposition time of 30 min was chosen based on the NiP coating morphology (Figure S2, Supporting Information) from a study of 15, 30, 45, and 60 min. Samples were thoroughly rinsed with DI water afterward.

*Electrodeposition of NFNS Active Layer*: NFNS active layer preparation was adapted from an optimized method used by Lu and Zhao.[[qv: 17b]] Nickel (II) nitrate hexahydrate (Ni(NO_3_)_2_·6H_2_O, 290.79 g mol^−1^), iron (III) nitrate nonahydrate (Fe(NO_3_)_3_·9H_2_O, 404.00 g mol^−1^) were purchased from Sigma Aldrich and as used. 3 × 10^−3^ m of Fe and Ni nitrates were prepared respectively by dilution in DI water and then thoroughly mixed to form a 6 × 10^−3^ m resultant mixture. Electrodeposition and subsequent electrochemical tests were done using Bio‐Logic VMP3 potentiostat with a standard three electrode electrochemical setup. The metal nitrate mixture was used as the electrolyte in the cell. Electrodeposition was carried out at a constant potential of −1 V with respect to Ag/AgCl electrode for 5 min with the deposition targets as the anode.

*Electrochemical Tests*: Electrolyte used was KOH (1 m, aq) (pH = 13.97). Linear sweep voltammetry (LSV) with ohmic drop correction (85%) was used for all OER and HER characterizations with a parallel placed Pt plate counter and as‐prepared samples as the working electrode. A Hg/HgO reference electrode was used, given the alkaline conditions. All electrochemical relationships were corrected to the reversible hydrogen electrode (RHE) to eliminate pH effects via the Nernst equation: *E*_RHE_ = *E*_recorded_ + 0.059 pH + *E*_reference_.

*Characterizations*: The transport properties of samples were characterized by Quantum Design physical property measurement system with temperature capabilities ranging from 2 to 300 K. SEM and TEM imaging and analyses were performed on Zeiss Supra 40 field‐emission SEM and Jeol 2010F TEM, respectively. XPS and XRD analyses were done using Axis Ultra DLD X‐ray photoelectron photospectrometer and Bruker D8 advanced diffractometer for chemical state and crystallinity investigations. Contact angle measurement was carried out using video contact angle Optima. Micro‐computed tomography (µCT) was performed using Caliper Life Sciences Quantum Fx µCT scanner for study of coating uniformity; sliced images were post‐processed using Image J. Fast‐speed video recording for water‐splitting studies was taken using the Photron Fastcam Mini AX200 camera.

## Conflict of Interest

The authors declare no conflict of interest.

## Supporting information

Supplementary

Supplementary

Supplementary
