# Supplementary material for: Metallization of 3D Printed Polymers and Their Application as a Fully Functional Water‐Splitting System
Source: Adv Sci (Weinh). 2019 Jan 24;6(6):1801670. doi: 10.1002/advs.201801670 (PMC6425437; doi:10.1002/advs.201801670)
Supplement: Supplementary file 1 — Supplementary [file ADVS-6-1801670-s001.pdf]

Copyright WILEY-VCH Verlag GmbH & Co. KGaA, 69469 Weinheim, Germany, 2018.

ADVANCED SCIENCE
Open Access

Supporting Information

for *Adv. Sci.,* DOI: 10.1002/advs.201801670

Metallization of 3D Printed Polymers and Their Application as a Fully Functional Water-Splitting System

Xinran Su, Xinwei Li, Chun Yee Aaron Ong, Tun Seng Herng, Yanqing Wang, Erwin Peng, and Jun Ding**

Supporting Information

Metallization of 3D printed polymers and their application as a fully functional water splitting system

Xinran Su, Xinwei Li, Chun Yee Aaron Ong, Tun Seng Herng, Yanqing Wang, Erwin Peng, Jun Ding**

X. Su, X. Li, Dr. C. Y. A. Ong, Dr. T. S. Herng, Dr. Y. Wang, Dr. E. Peng, Prof. J. Ding
Department of Materials Science and Engineering, National University of Singapore, 117576, Singapore
* Corresponding Authors

E-mail: mseer@nus.edu.sg, msedj@nus.edu.sg

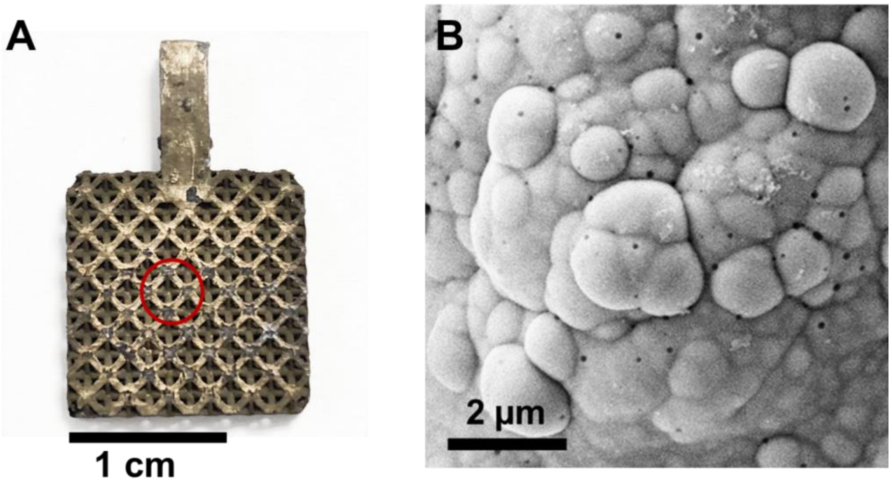

Supporting Figure S1. NiP@Truss sample prepared without addition of SDS surfactant during electroless plating. a) Digital image of the sample, with red circle inserted to highlight the poorly coated region coming from polymer hydrophobicity and lattice bubble-trapping effects. b) SEM image of coated area, with pits distributed across the non-uniform NiP coating. As surfactant is shown to play an important role in cleaning up H_2 bubbles from the plating substrate, [1] the elimination of SDS resulted in such an observation of the pits.

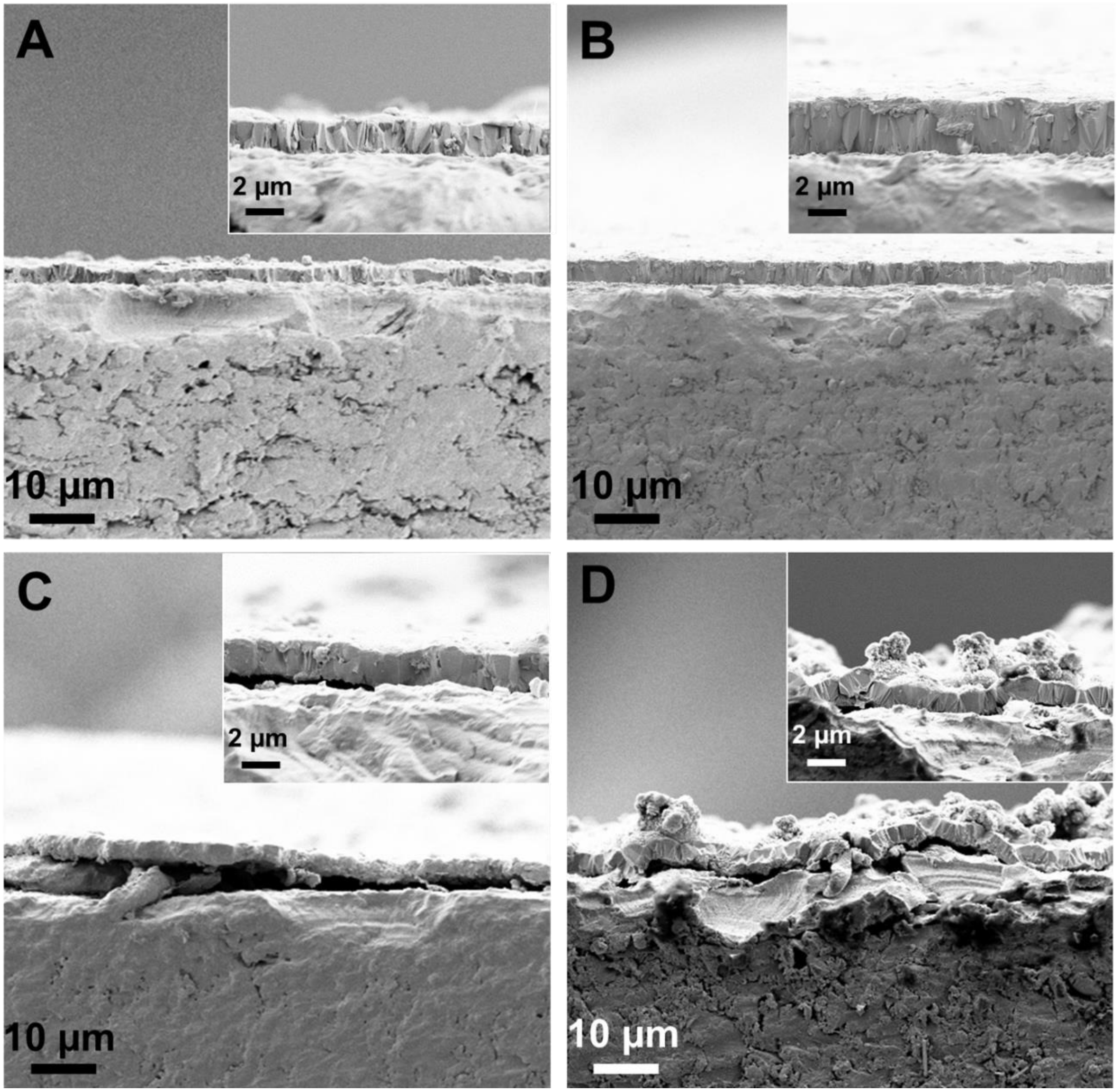

Supporting Figure S2. NiP coating times of a) 15 min, b) 30 min, c) 45 min and d) 60 min. 30 min was found to be the ideal condition with a sufficiently thick and well adhered NiP layer of 2.7 µm. Below 30 min, thickness is insufficient and will lead to a higher electrical resistance. Above 30 min, up to 45 min and 60 min, no improvements in NiP thickness was observed but however with increasing film disorder, poor adhesion, significant detachments and balling effects.

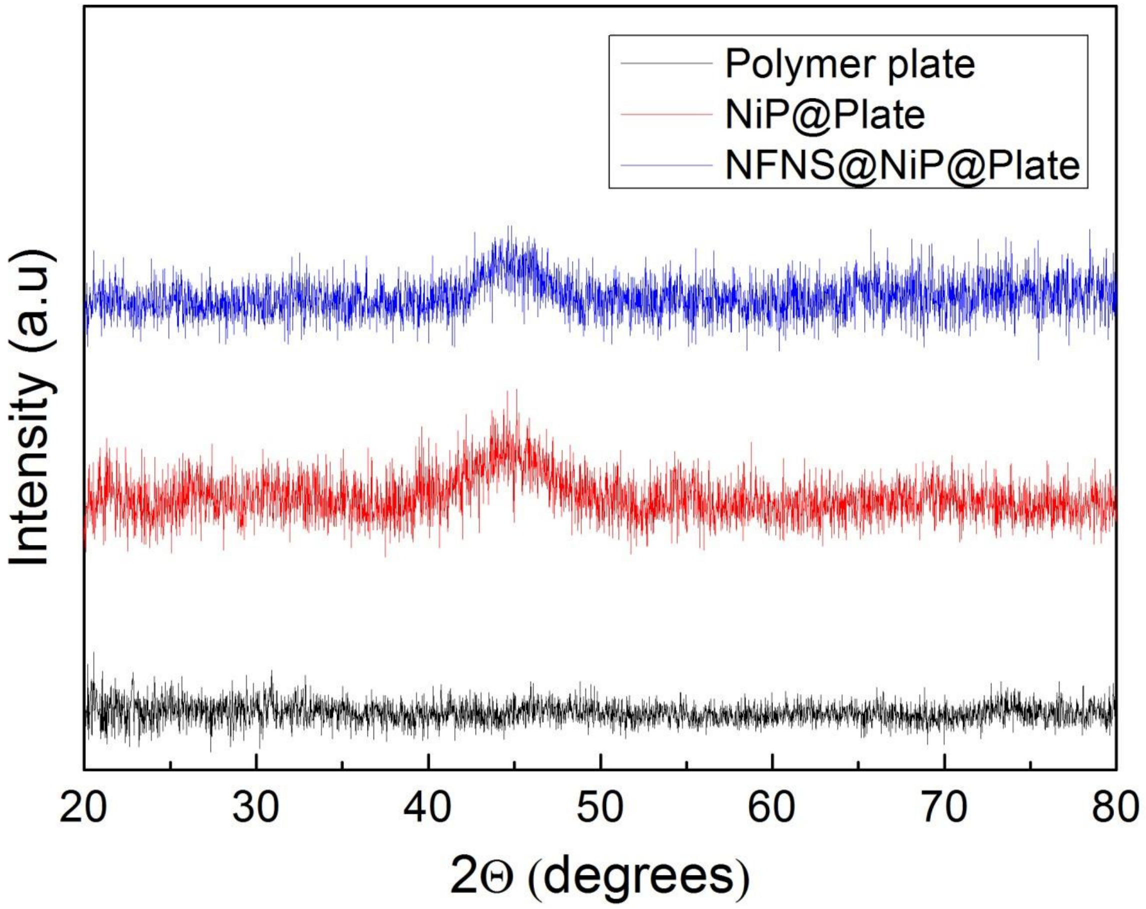

Supporting Figure S3. XRD patterns of polymer plate, NiP@Plate and NFNS@NiP@Plate, revealing all to be of amorphous nature.

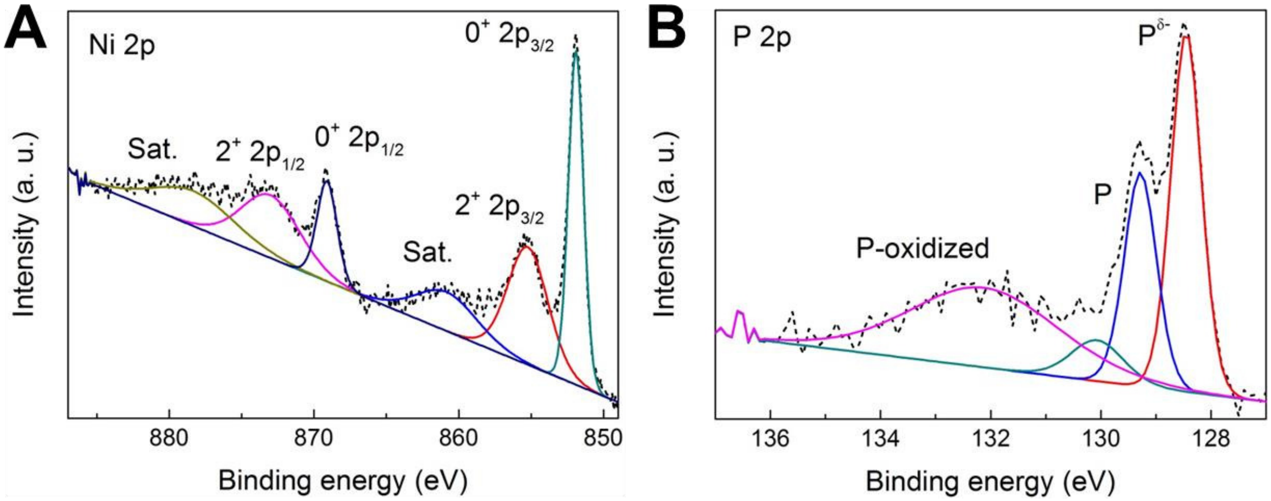

Supporting Figure S4. XPS peak fittings of a) Ni 2p, b) P 2p.

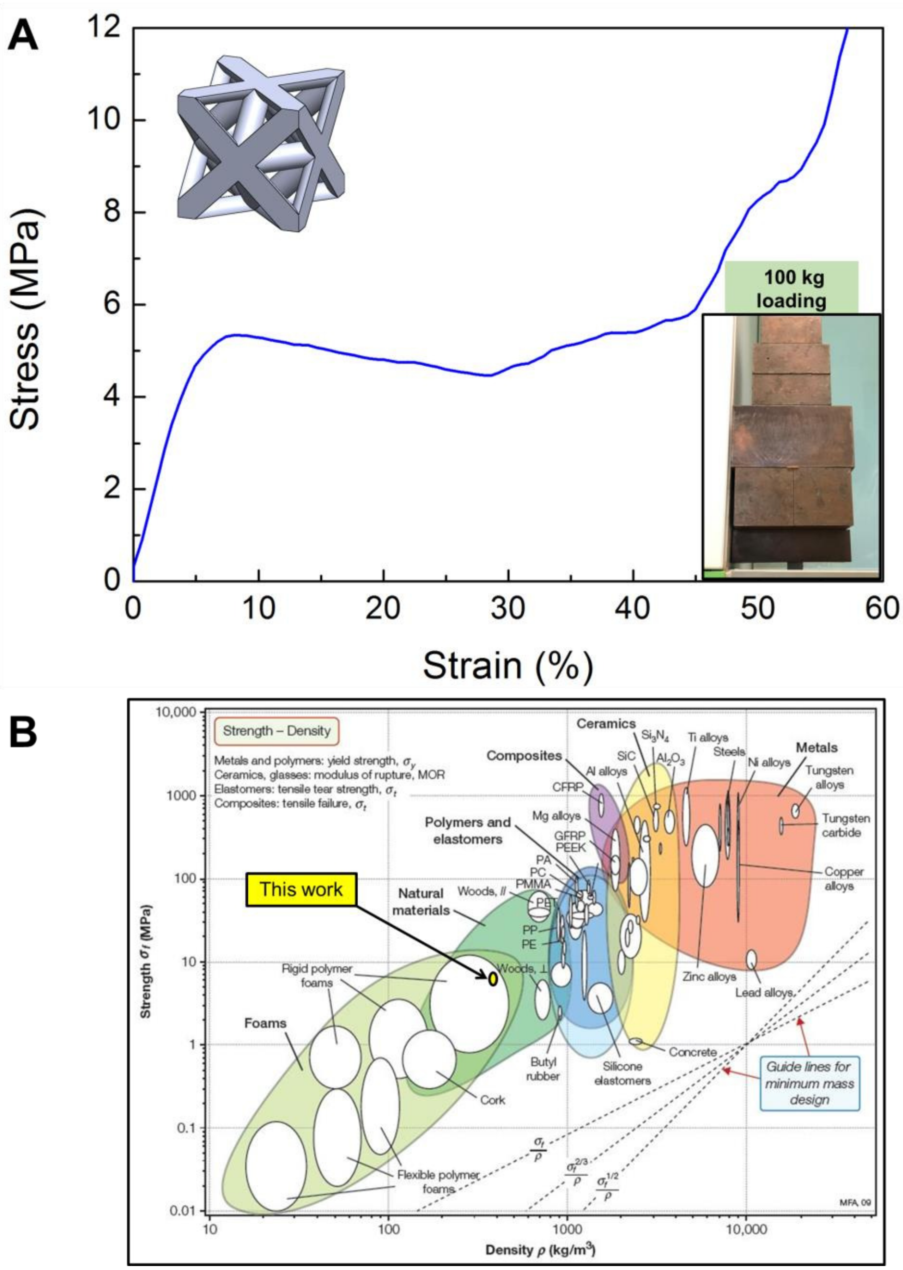

Supporting Figure S5. a) Representative stress-strain curve of the 3D printed polymeric octet- truss under compression; insert shows a computer-aided design representation of a single unit cell and a digital image showing 100 kg metal loading on it. b) Ashby plot of strength vs density of materials, the 3D printed octet-truss in this work is amongst the strongest rigid polymer foams. Ashby plot reproduced from.[2]

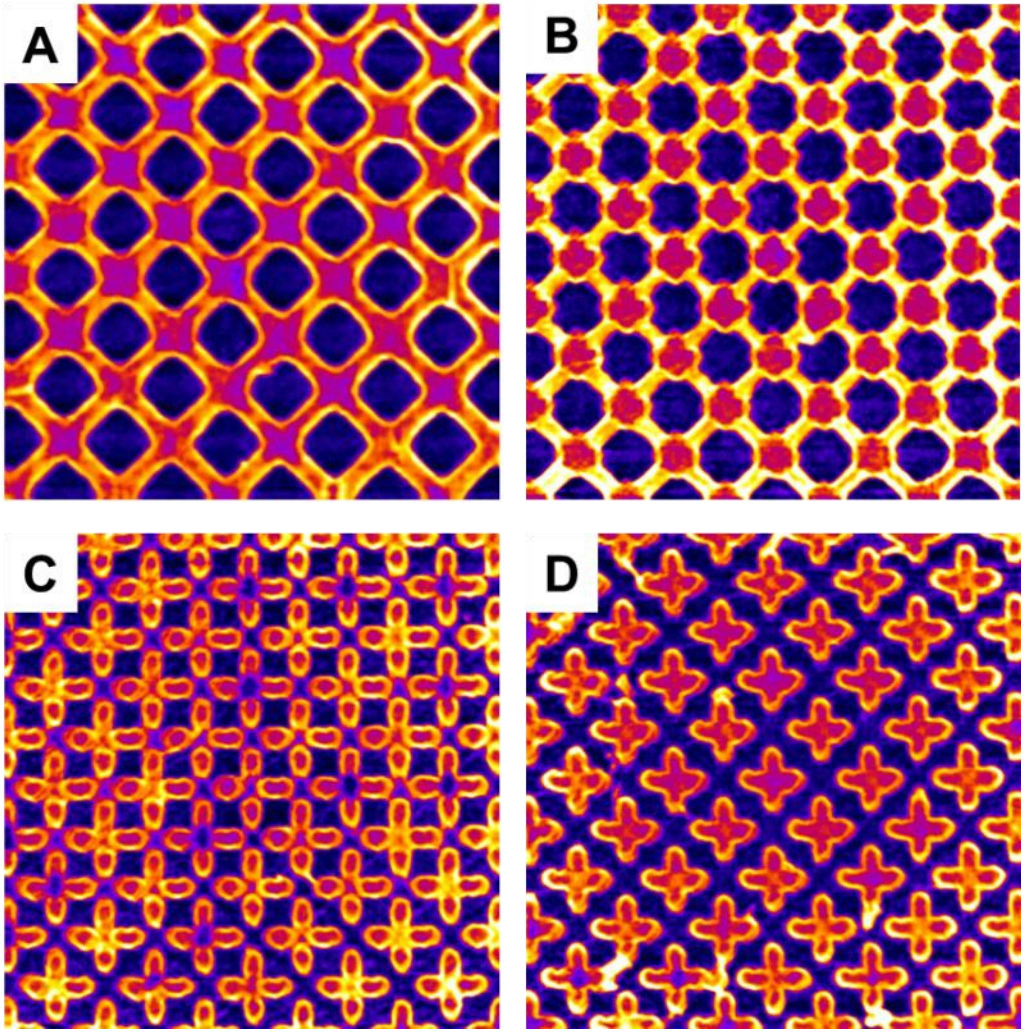

Supporting Figure S6. a-d) X-ray computer-tomography (CT) of the octet truss electrode at different locations; color contrast due to different signal intensity from a metal and polymer reveals the uniform coating on both the exterior and interior of the electrode: NiP (yellow) and polymer (tending towards red). Due to limitations of the X-ray technique and post-processing, color contrast should be taken as a representation only and not an accurate depiction of NiP plated thickness or dimensions.

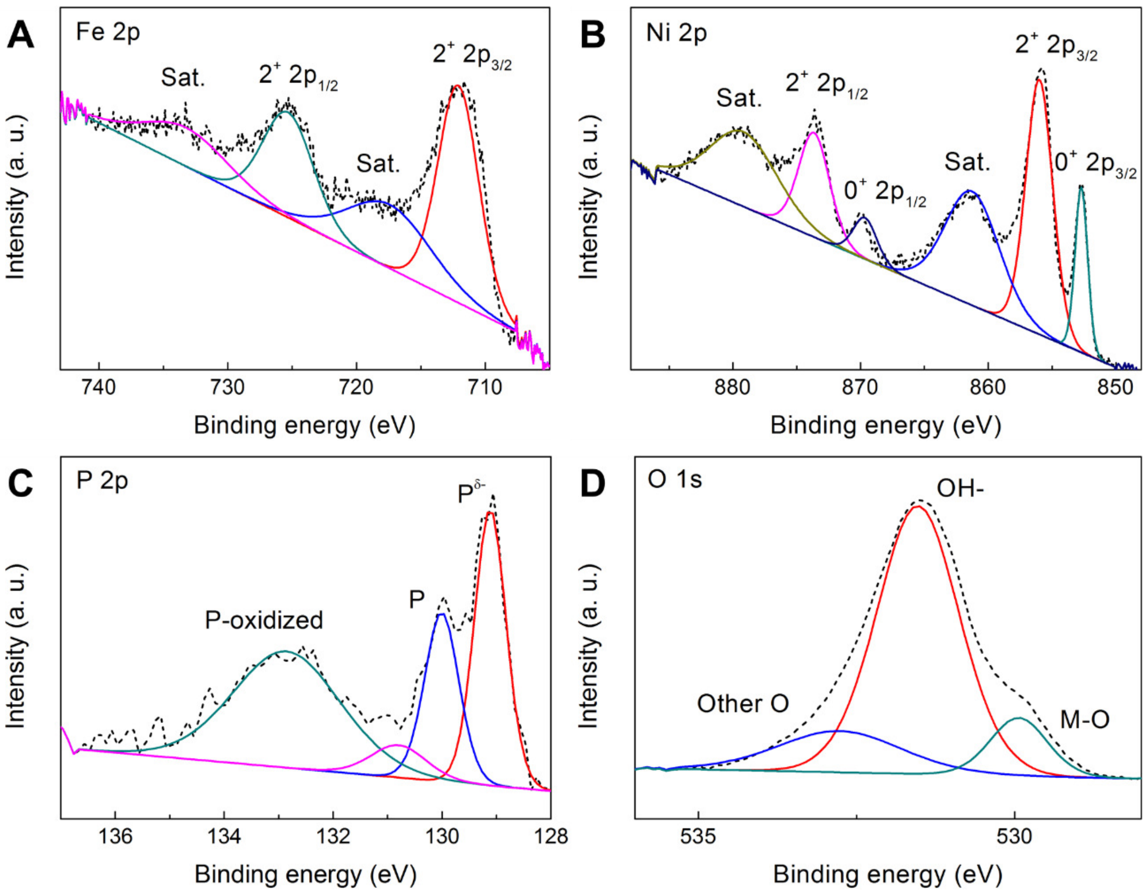

Supporting Figure S7. XPS peak fittings of a) Fe 2p, b) Ni 2p, c) P 2p and d) O 1s.

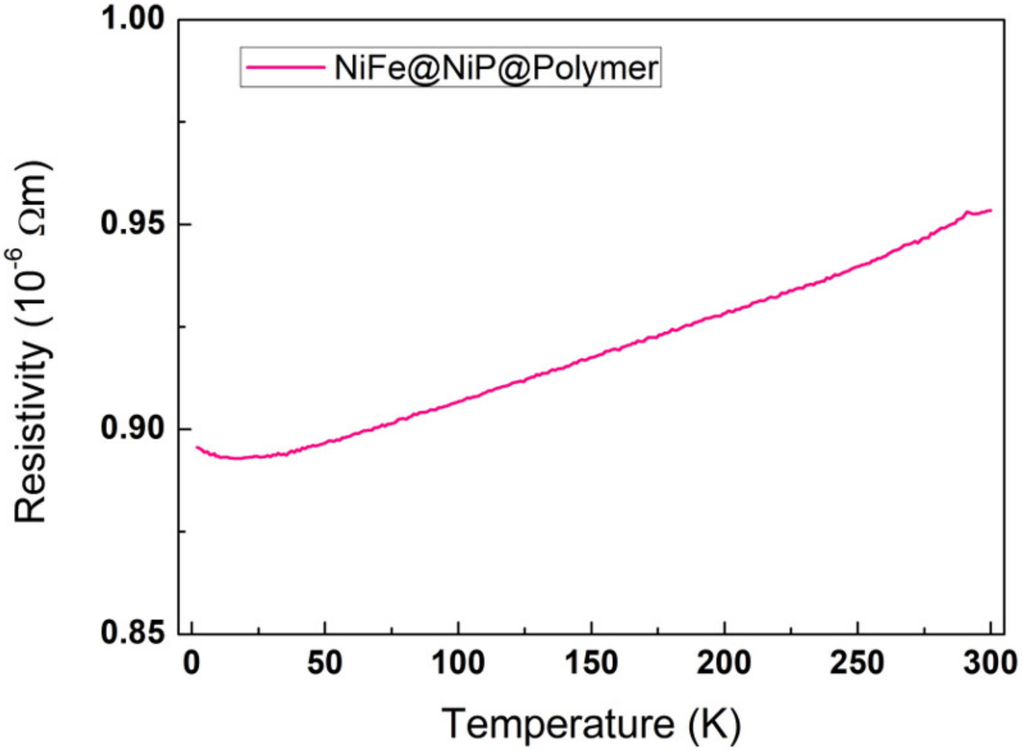

Supporting Figure S8. Measured resistivity values of NFNS@NiP@Polymer across a range of temperatures from 3 K to 300 K.

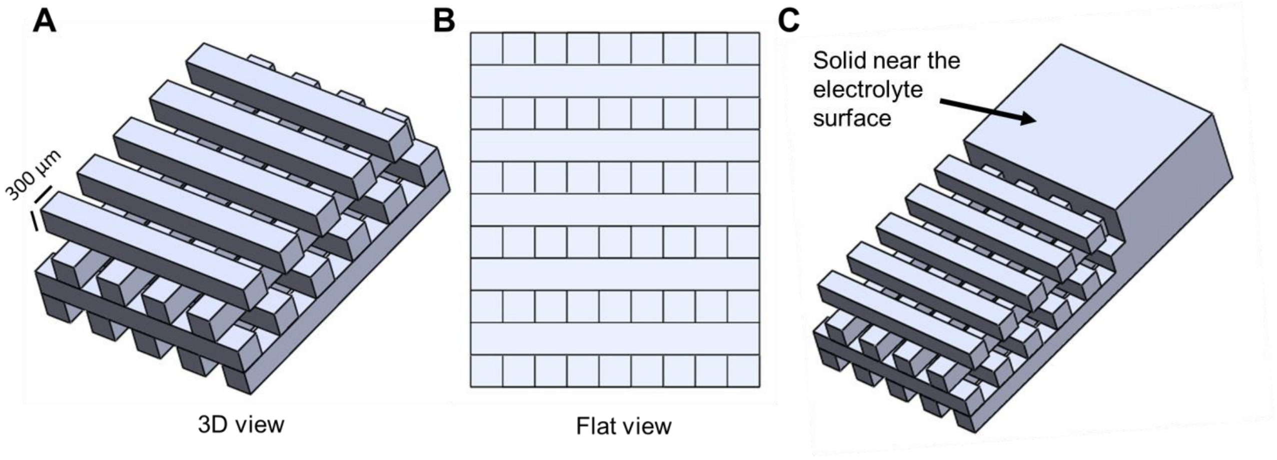

Supporting Figure S9. Computer-aided design model of a representative small part of the gas-bubble separating mesh. Design principle based on maximizing the surface area that directly blocks off oncoming bubbles while still porous enough to allow low electrolyte resistance increase. a) 3D view, illustrating the alternating wood-pile structure configuration and its associated b) top view, illustrating the maximum surface area; c) Improved design with the top portion being made fully solid, to fully impede bubble exchanges.

Table S1. Summary of the surface area metrics of various lattices of 500 um strut and 37.89 % volume fraction (that corresponds to octet with 500 um struts).

Architecture	Surface area of designed electrode (mm^2)	Normalized w.r.t fully solid electrode	Unit cell representation
Octet-500(37.89%)	3076.48	4.1	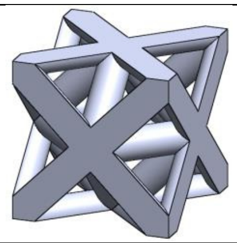
BCC-500	1484.21	1.98	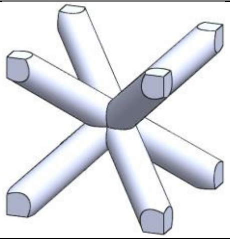
BCC-37.89%	1898.78	2.53	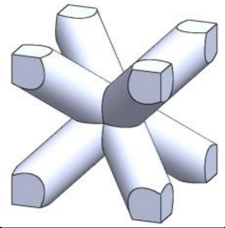
Cubic-500	965.48	1.29	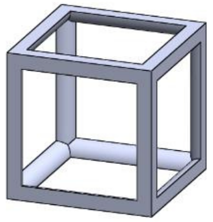
Cubic-37.89	1694.89	2.26	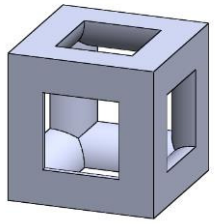

RD-500	2439.38	3.25	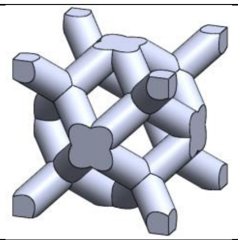
RD-37.89%	2530.34	3.37	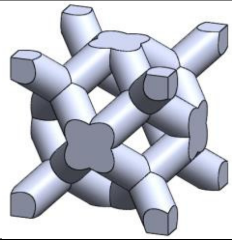
Full solid	750*<br><br>*For a 15 mm square faced electrode, total surface area = 2*15*15 (faces) + 4*5*15 (sides) = 750 mm^2	1	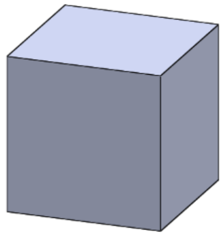

Table S2. Comparison of the OER performance of NFNS@NiP@Truss with current literature on Ni and Fe associated and other 3D printed catalysts. Our reported value showed an excellent combination of lower overpotential and Tafel slope, with overpotential surpassing most reported by current works.

Catalyst and its substrate	Overpotential at 10 mA cm^{-2} (mV)	Tafel slope (mV dec^{-1})	Working Condition	Ref
**Ni + Fe based**				
NiFe-LDH[a] on NF	215	28	KOH (1M, aq)	[3]
NiFe-LDH[a] array on NF	224	44	KOH (1M, aq)	[4]
NiFe on NF[b]	210	-	KOH (1M, aq)	[5]
NiFe-LDH/CNT on GC[c]	247	31	KOH (1M, aq)	[6]
NiFe/rGO-LDH on NF[b]	206	39	KOH (1M, aq)	[7]
Ni_2Fe_1 nanofoam	190	70	KOH (1M, aq)	[8]

**3D printing based**				
IrO_2 on 3D printed stainless steel gauze	~ 350	92	KOH (1M, aq)	[9]
IrO_2 on 3D printed stainless steel ribbon	370	-	KOH (0.1M, aq)	[10]
3D printed porous stainless steel	270	43	KOH (1M, aq)	[11]
**NFNS@NiP@Truss**	**197**	**51**	KOH (1M, aq)	**This work**

Notes: [a]LDH = layered double hydroxide; [b]NF = nickel foam; [c]GC = glassy carbon

Movie S1: Video shows an extract of about 10 cycles of compressions on a NiP plated flexible polymer. Excellent adhesion was observed with no NiP peeling off and LEDs remaining lit. In addition, NiP film was also shown to be ductile enough to allow the polymer to be still flexible and yet itself not cracking up (at least from the macro point of view given that the structure is still conductive enough). It is worth noting that the brightness changes to the LED lights may be due to contact issues during the compressions. Second part of the video shows that even after multiple very harsh compressions (not shown), the NiP layer is still intact even though the polymer had already failed, ie. struts are broken.

Movie S2: In this video, the stepping of an octet truss configuration (strut diameter to cell length ratio of 1:5) that was used in Figure 1d and water splitting electrodes was demonstrated. With a cross-sectional area of 225 mm2, given a yielding stress of about 5 MPa, it means that such a structure can withstand about 110 kg (1100 N) of weight before any failure initiates. As such, we illustrate it robustness with the compression by a person of > 70 kg. Weighing scale is used for clear illustration. The full body weigh was applied to the lattice, ie. no part of the show touches the weighing scale. No damages were observed to the lattice after stepping.

References

[1] B.-H. Chen, L. Hong, Y. Ma, T.-M. Ko, Industrial & engineering chemistry research 2002, 41, 2668.

[2] M. F. Ashby, Materials Selection in Mechanical Design, Elsevier, United States **2010**.

[3] X. Lu, C. Zhao, Nature communications **2015**, 6.

[4] Z. Li, M. Shao, H. An, Z. Wang, S. Xu, M. Wei, D. G. Evans, X. Duan, Chemical science **2015**, 6, 6624.

[5] J. Luo, J.-H. Im, M. T. Mayer, M. Schreier, M. K. Nazeeruddin, N.-G. Park, S. D. Tilley, H. J. Fan, M. Grätzel, Science **2014**, 345, 1593.

[6] M. Gong, Y. Li, H. Wang, Y. Liang, J. Z. Wu, J. Zhou, J. Wang, T. Regier, F. Wei, H. Dai, Journal of the American Chemical Society **2013**, 135, 8452.

[7] X. Long, J. Li, S. Xiao, K. Yan, Z. Wang, H. Chen, S. Yang, Angewandte Chemie **2014**, 126, 7714.

[8] S. Fu, J. Song, C. Zhu, G.-L. Xu, K. Amine, C. Sun, X. Li, M. H. Engelhard, D. Du, Y. Lin, Nano Energy 2018, 44, 319.

[9] A. Ambrosi, M. Pumera, Advanced Functional Materials **2017,** 28, 1700655.

[10] A. Ambrosi, J. G. S. Moo, M. Pumera, Advanced Functional Materials **2016**, 26, 698

[11] X. Huang, S. Chang, W. S. V. Lee, J. Ding, J. M. Xue, Journal of Materials Chemistry A **2017**, 5, 18176
